# Periscope of Dermatology

**Published:** 1894-11-10

**Authors:** 


*(To be continued.)*

### PERISCOPE OF DERMATOLOGY.

**Thyroid Feeding.**—Bramwell[1] read another important paper on this subject before the British Congress of Dermatology, According to him, the beneficial action of the drug consists in an improvement in nutrition of the skin brought about by an invigorated condition of the nervous system. He therefore recommends it in cases of skin disease, which appear to depend on defects in innervation, such as Morvan's disease, sclerodactylia, &c.

He gives details of twenty cases of psoriasis treated by thyroid feeding, and states, as a result of his experience, that it is the most valuable internal remedy which has yet been discovered for this disease, and that in a considerable number of cases no other treatment is necessary. In lupus he finds that thyroid feeding produces considerable improvement, and in cases of icthyosis, eczema, pemphigus, &c., it is also beneficial. Referring to the unpleasant effects of the drug, he states that the quantity required to produce distinct symptoms of thyroidism varies in different persons. He has observed very rapid action of the heart, and occasionally suppurative lesions as a result of an over-dose, but never albuminuria nor glycosuria. When the treatment is to be continued for a long time, as in cases of lupus, small doses should be given; but in cases of psoriasis large doses are required and are well borne. He prefers the dry tabloids. Jackson,[2] besides employing thyroid feeding in three cases of myxœdema, gave it in a case of icthyosis and in one of dermatitis exfoliativa without any marked improvement. It was the opinion of the American Dermatological Association that the number of good results in cases of psoriasis reported in England were due probably to the indoor hospital treatment which these patients received at the same time, and that the remedy itself was not really worth employing.

**Lupus.**—During a discussion on lupus at the Edinburgh Medical and Surgical Society[3] Miller spoke in favour of Bier's congestive method of treatment combined with scraping. He holds that the congestion produced prevents the tendency of the disease to spread. Jamieson urged the use of pure carbolic acid, and the fine pointed thermo-cautery in severe cases. Bell, on the other hand, holds that Lupus ought to be treated like cancer, heroically and early. Bidwell[4] recommends free excision combined with Thiersch's skin-grafting. He gives results of eight cases so treated, and states that the resulting scar is satisfactory. Under the name of scrofuloderma verrucosa, Namach[5] describes a case of multiple lupus occurring in a child aged seventeen months. The eruption consisted of irregular-shaped, variously-sized patches covered with thick darkish crusts, which were situated over the face, neck, belly, legs, and buttocks. On removing the crusts the surface had a verrucous appearance, but was painless and did not itch. Von Hoorn[6] records the effect of injections of thiosinamin in lupus. There was local reaction of redness, and swelling, together with a feeling of heat and tension, which was followed by desquamation. In severe cases there was more marked improvement than in slight ones. Local treatment should be employed at the same time. Payne[7] in a clinical lecture on lupus erythematosus, reviews all the older opinions on this subject. He considers that the disease originates in a circulatory disturbance, but subsequently develops properties distinguished from a mere inflammatory condition. He draws attention to the fact that the scars, though always present, are never deep. The commonest age at which the disease appears is between the ages of 20 and 50, but its connection with other forms of tuberculosis is rare. Histologically he has never seen giant cells or tubercle bacilli, and, on account of the failure to produce tuberculosis in the lower animals by experimental inoculation, he considers that the disease is not tuberculous in origin. Since the disease is to be considered of life-long duration, it is very difficult to produce any but a temporary improvement by treatment. The progress of the disease is affected not so much by the general nutrition of the body as by the state of the circulation. He therefore places great faith in quinine, which should be given in daily does of from 10 to 30 grains. This will not produce unpleasant symptoms, as these patients bear quinine well. Locally he applies cold lead lotion with glycerine in mild cases, while in others he prefers a solution of salicylic acid in collodion; in severe cases where more energetic treatment is necessary he finds the best results from thorough linear scarification together with the use of iodoform. Finally he quotes cases where he considers the improvement has been due to quinine. Leredde[8] describes the case of a woman affected with characteristic and typical lupus erythematosus on the face, who in consequence developed considerable cervical adenopathy. The glands suppurated and were opened; numerous tubercle bacilli were found in the pus. This case, he thinks, offers unimpeachable evidence of the dependence of lupus erythematosus on the tubercle bacillus.

**Pemphigus.**—Stopford Taylor[9] records two fatal cases of pemphigus. His first case, a woman aged forty-four years, had enjoyed good health until the development of the disease; she had a typical eruption, but died comatose fifteen days after the commencement of the attack; there was no albuminuria nor glycosuria. The second case, a woman aged forty-five years, had suffered for thirteen months from sores on the head and from ulcerated throat; subsequently the nose and mouth became sore. When first seen, there were broken down bullæ and crusts on the head; the hard and soft palates and buccal mucous membranes were completely denuded of epithelium; the general condition was feeble. In spite of liberal diet and stimulants, her condition did not improve, but fresh bullæ appeared in different parts, and there was continued bleeding from the exposed mucous surfaces. She became delirious and died from exhaustion. The author remarks that he has never before met with two similar cases; he considers that the second case is an example of the malignant variety of the disease described by Neuman under the name of pemphigus vegetans. A course of irritation of more or less lengthy duration is requisite for the development of such a disease. Sansom[10] describes the case of a girl aged twelve who presented ecchymoses about the eyelids, oozing from the mouth, and hæmorrhagic spots and stains on the skin of the abdomen, chest, back, and extremities; at the same time bullæ containing deeply blood-stained fluid, appeared on the alæ nasi, the abdomen, and the extremities. There was hæmatemisis and melæna followed by hæmorrhages into both optic discs. The patient was treated with thirty-grain doses of sulpho-carbolate of soda every four hours, and left the hospital quite cured in 35 days. He regards this case as one of purpura hæmorrhagic, combined with acute pemphigus. Langfeldt[11] describes three cases of bullous eruption which persisted for about two months in spite of treatment. In the first case a bulla appeared on a woman's hand in consequence of the sting of a fly. After a few days the whole arm was covered with bullæ. A week later a carpenter had a similar attack of bullæ near a severe wound on the leg; a neighbour, too, also had a similar eruption. He urges that all cases of bullous eruptions resembling pemphigus should be carefully observed and reported.

[1] Brit. Journ. of Derm., July, 1894, p. 193; and New York Med. Journ., Aug. 4, 1894, p. 157. [2] Med. Rec., June 16, 1894, p. 763. [3] Ed. Med. Journ., Aug., 1894, p. 159. [4] Lancet, July 21, 1894, p. 130. [5] New York Med. Journ., July 28, 1894, p. 116. [6] Brit. Med. Journ., Ep., July 7, 1894, p. 3. [7] Clin. Journ., Aug. 1, 1894, p. 223. [8] Med. Week, June 22, 1894, p, 297. [9] Brit. Journ. of Derm., June, 1894, p. 177. [10] Med. Week, June 1, 1894, p. 255. [11] Am. Med. and Surg. Bull, June 1, 1894, p. 693.

(*To be continued.*)

